# Systematic analysis on multiple Gene Expression Omnibus data sets reveals fierce immune response in hepatitis B virus‐related acute liver failure

**DOI:** 10.1111/jcmm.15561

**Published:** 2020-07-19

**Authors:** Huadi Chen, Wenting Zhao, Yixi Zhang, Yiwen Guo, Weixin Luo, Xiaobo Wang, Yu Nie, Maodong Ye, Changjun Huang, Dongping Wang, Maogen Chen, Xiaoshun He, Qiang Zhao

**Affiliations:** ^1^ Organ Transplant Center, The First Affiliated Hospital Sun Yat‐sen University Guangzhou China; ^2^ Guangdong Provincial Key Laboratory of Organ Donation and Transplant Immunology Guangzhou China; ^3^ Guangdong Provincial International Cooperation Base of Science and Technology (Organ Transplantation) Guangzhou China; ^4^ School of Traditional Chinese Medicine Southern Medical University Guangzhou China; ^5^ Liver Transplantation Center Beijing Friendship Hospital Capital Medical University Beijing China

**Keywords:** bioinformatics analysis, HBV‐ALF, immune, inflammation, WGCNA

## Abstract

Acute liver failure (ALF) caused by hepatitis B virus (HBV) is common type of liver failure in the world, with high morbidity and mortality rates. However, the prevalence, genetic background and factors determining the development of HBV‐related ALF are rarely studied. In this study, we examined three Gene Expression Omnibus (GEO) data sets by bioinformatics analysis to identify differentially expressed genes (DEGs), key biological processes and pathways. Immune infiltration analysis showed high immune cells infiltration in HBV‐related ALF tissue. We then confirmed natural killer cells and macrophages infiltration in clinical samples by immunohistochemistry assay, implying these cells play a significant role in HBV‐ALF. We found 1277 genes were co‐up‐regulated and that 1082 genes were co‐down‐regulated in the 3 data sets. Inflammation‐related pathways were enriched in the co‐up‐regulated genes and synthetic metabolic pathways were enriched in the co‐down‐regulated genes. WGCNA also revealed a key module enriching in immune inflammation response and identified 10 hub genes, differentially expressed in an independent data set. In conclusion, we identified fierce immune inflammatory response to elucidate the immune‐driven mechanism of HBV‐ALF and 10 hub genes based on gene expression profiles.

## 1 INTRODUCTION

Acute liver failure (ALF) is a serious clinical syndrome. The survival prognosis of ALF is extremely poor with a high short‐term mortality. ALF is characterized by acute multiple system organ failure, jaundice, coagulation with ascites and/or encephalopathy in patients without chronic liver disease within 4 weeks.^1^ The incidence of hepatitis B virus (HBV) infection is high in China. There are 120 million carriers of HBV and 20 million patients with chronic hepatitis B.^2^ A study showed 0.1%‐0.5% HBV‐infected patients would develop ALF.^3^ HBV infection is a major risk factor for ALF patients. However, current treatments for HBV‐ALF, which include antiviral treatment and other support measures, are not satisfying except liver transplantation.^3^ But owing to dramatic clinical course and shortage of donor organ, many patients die during the waiting for the proper donor liver. In fact, the prognosis of acute liver failure mainly depends on early detection and intervention, and early diagnosis and timely intervention could help prevent or reverse the decompensation process.^4^, ^5^

At present, the pathophysiological mechanism of HBV‐ALF has not been clarified. An explanation, in detail, of the basic mechanism of HBV‐ALF is urgently needed. Nissim et al^6^ revealed hepatic stem/progenitor cells and fibrogenesis were positively correlated with the degree of liver necrosis in HBV‐ALF. Besides, several research groups reported that humoural immunity contributes largely to pathogenesis of HBV‐related ALF, which is reflected by plasma cells and complements accumulation in necrotic areas of HBV‐related ALF tissue.^7^, ^8^, ^9^ Previous studies have shown that bioinformatics analyses in cross‐sectional studies are very useful in identifying factors related to HBV‐related acute‐on‐chronic liver failure progression.^10^, ^11^ In this study, we performed a comprehensive bioinformatics analysis on multiple Gene Expression Omnibus data sets to reveal fierce immune inflammatory response and hub genes in HBV‐ALF.

## 2 MATERIALS AND METHODS

### 2.1 Data collection and processing

We downloaded expressing profiles of mRNA of HBV‐ALF and normal control from Gene Expression Omnibus (GEO) database (http://www.ncbi.nlm.nih.gov/geo/). Data sets, GSE14668, GSE38941 and GSE96851, were used to performed differentially expression analysis, immune infiltration analysis and construct co‐expression networks and identify hub genes in our study. Data set GSE62029 was selected to validate the hub genes identified in WGCNA. The raw data were downloaded and processed to normalize in R software (ver. 3.6.1). Limma package was used to screen for differentially expressed genes(DEG).^12^ We identified DEGs with a |log_2_ fold change (FC)| > 1 and adjusted *P* value < .05.

### 2.2 Enrichment analysis

The clusterProfiler R package^13^ was used for functional enrichment analysis, including Gene Ontology (GO) and Kyoto Encyclopedia of Genes and Genomes (KEGG) pathway analysis. GO enrichment includes cell components (CC), biological processes (BP) and molecular functions (MF). A term was considered statistically significant if adjusted *P* value < .05.

### 2.3 Protein‐protein interaction (PPI) network analysis and genes clusters identification

The co‐expressed genes with a |log_2_ FC| > 3 and adjusted *P* value < .01 in GSE14668, GSE38941 and GSE96851 datasets were uploaded to STRING database to get the PPI diagram. The Cytoscape software v.3.7.1 was used to construct PPI network based on STRING analysis result. The Molecular Complex Detection (MCODE) plug‐in^14^ was used to identify gene clusters. The genes cluster with the highest score was further analysed by biological processes enrichment.

### 2.4 Immune infiltration examination

We combined GSE14668, GSE38941 and GSE96851 data sets by applying combat method in the sva R package^15^ to remove batch effects. The combining expression matrix was used for further immune infiltration analysis. We uploaded the combining expression matrix to TIMER2^16^ database to calculate immune infiltration and the result of infiltration examination was downloaded. We showed the absolute abundance of immune cells estimated by the MCP‐counter method from the MCP‐counter package^17^ in this study. GSVA R package^18^ was used to calculate the ssGSEA scores of four hallmark cytokine signal pathways.

### 2.5 Immunohistochemistry

Paraffin‐embedded and formalin‐fixed HBV‐ALF and healthy donor liver samples were cut into 5‐μm sections, which were processed for immunohistochemistry. The sections were incubated with antibody against CD68 (1:100, ZSJQB, ZM‐0060) and CD57 (1:200, ZSJQB, ZM‐0058). The numbers of CD68+ macrophage and CD57+ natural killer (NK) cell were manually counted under a high‐power field (200×). Two independent observers evaluated the immunohistochemical variables.

### 2.6 Weighted gene co‐expression network analysis (WGCNA) and identification of hub modules as well as genes

The genes ranking the first 5000 of median absolute deviation in the combining expression matrix were kept for WGCNA by ‘WGCNA’ package in R.^19^ We used unsigned type of topological overlap matrix with a power β of 14 to construct WGCNA network and detect modules. Correlation between module eigengenes and acute liver failure was calculated to identify the relevant module. In addition, we calculated gene significance (GS) of all genes and defined the average GS for all the genes as module significance in a module. The modules with the highest average GS were considered as the hub modules and the top ten GS genes in the hub modules were regarded as hub genes. Moreover, the hub genes were selected for validation in GSE62029.

### 2.7 Ethics statement and ethical approval

This study was approved by the Research Ethics Committee of the First Affiliated Hospital of Sun Yat‐sen University (No. 201819). All the experiments were conducted in accordance with the guidelines approved by the First Affiliated Hospital of Sun Yat‐sen University. All procedures in the study involving human participants met the ethical standards of the Ethics Committee of the First Affiliated Hospital of Sun Yat‐sen University. All the livers were procured from the Organ Transplantation Center of the First Affiliated Hospital of Sun Yat‐sen University. All the individual participants included in this study provided informed consent.

### 2.8 Statistical analysis

All statistical analyses were performed in R software (Version 3.6.1). The Wilcoxon rank sum test was used to compare the difference of two groups of quantitative data. All *P* values were bilateral, and *P* < .05 was defined as statistically significant.

## 3 RESULTS

### 3.1 Study design

**Figure 1:**
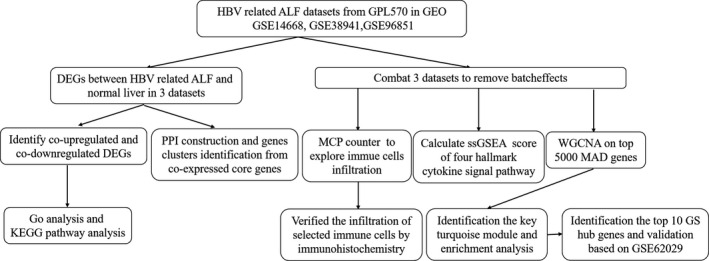
Flow diagram of the study design. ALF, acute liver failure; DEGs, differentially expressed genes; GEO, gene expression omnibus; Go, gene ontology; GS, gene significance; HBV, hepatitis B virus; KEGG, Kyoto Encyclopedia of Genes and Genomes; MAD, median absolute deviation; MCP, microenvironment cell populations; PPI, protein‐protein interaction; ssGSEA, single‐sample gene set enrichment analysis; WGCNA, weighted gene co‐expression network analysis

The flow chart of this research design is shown in Figure 1. Our main objective was to identify the core genes, pathways and immune infiltration involved in the development of ALF. Firstly, we used three GEO data sets (GSE14668, GSE38941 and GSE96851) in this study. We extracted the gene expression data of HBV‐ALF and normal liver tissues to determine the DEGs between the two histological/diagnostic types. Based on these DEGs, the co‐expressed DEGs were identified. Then, the biological functions, KEGG pathways and PPI were analysed. Besides, we combined three data sets by combat method using sva R package to further MCP‐counter and ssGSEA analysis. Selected immune cells infiltration was verified by immunohistochemistry. At last, WGCNA was performed on the top 5000 median absolute deviation genes from the combining expression matrix. A key turquoise module and top 10 GS hub genes were identified. Moreover, the hub genes were validated significant up‐regulation in HBV‐ALF tissues based on an independent data set GSE62029.

### 3.2 Main characteristics of samples in the four datasets

GSE14668, GSE38941, GSE96851 and GSE62029 from the GEO database were examined in our study. All data sets were processed in R software. Table 1 showed the diagnostic classification and number of samples contained in each data set include in the present study. GSE14668 included 8 HBV‐ALF tissues and 20 normal liver tissues. GSE38941 contained 17 HBV‐ALF tissues and 10 normal liver tissues. GSE96851 comprised 17 HBV‐ALF tissues and 17 normal liver tissues. The above data sets were removed batch effects by combat method to make up the combining expression matrix, which included 42 HBV‐ALF tissues and 47 normal liver tissues therefore. GSE62029 consisted of 13 HBV‐ALF samples and 17 normal samples.

**Table 1:** The samples from four data sets included in the study

GEO_ID	HBV‐ALF	Normal	Platform
GSE14668	8	20	CPL570
GSE38941	17	10	CPL570
GSE96851	17	17	CPL570
GSE62029	13	17	CPL570

Abbreviations: GEO, gene expression omnibus; HBV‐ALF, hepatitis B virus‐related acute liver failure.

### 3.3 DEGs between HBV‐ALF and normal liver tissues

**Figure 2:**
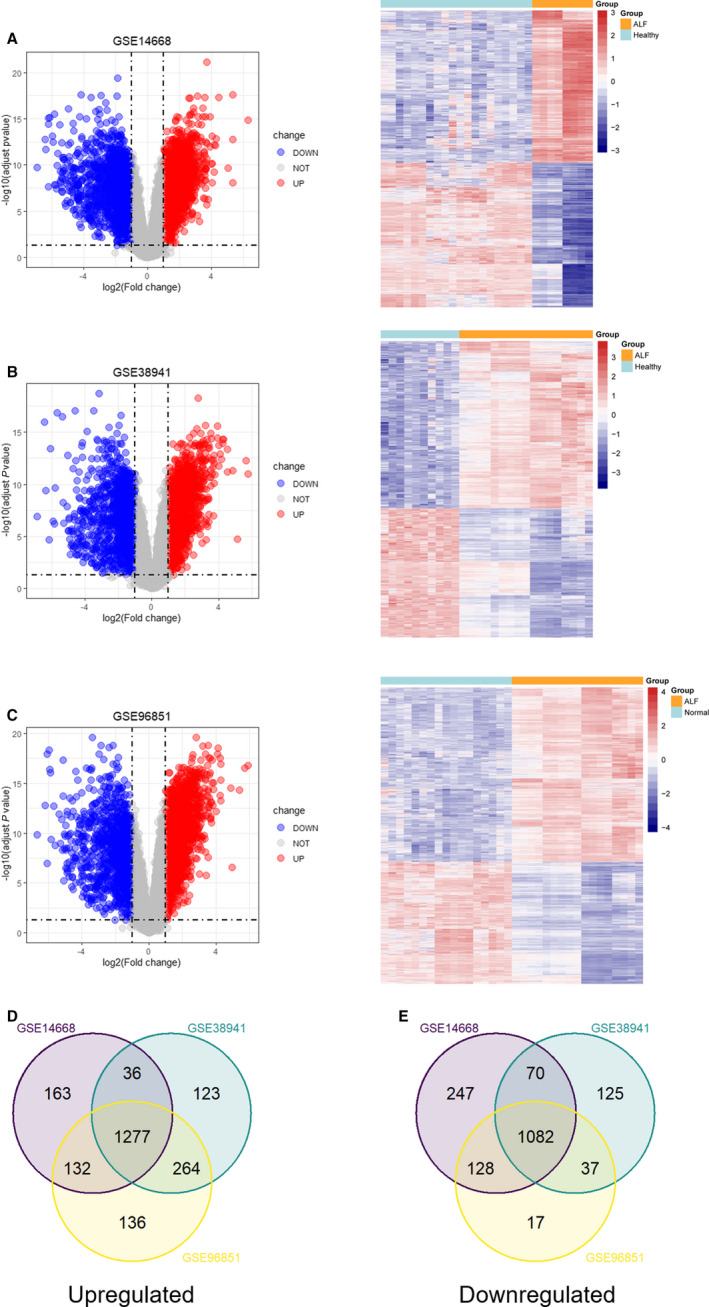
Volcano plot and heat maps for potential DEGs between HBV‐ALF and normal liver tissues in 3 data sets. A, Potential DEGs in GSE14668 (containing 8 HBV‐ALF tissues and 20 normal liver tissues). 1608 genes were up‐regulated and 1527 genes were down‐regulated. B, Potential DEGs in GSE38941 (containing 17 HBV‐ALF tissues and 10 normal liver tissues). 1700 genes were up‐regulated and 1314 genes were down‐regulated. C, Potential DEGs in GSE96851 (containing 17 HBV‐ALF tissues and 17 normal liver tissues). 1809 genes were up‐regulated and 1264 genes were down‐regulated. D and E, The Venn diagram shows the co‐expressed genes among GSE14668, GSE38941 and GSE96851. 1277 co‐up‐regulated genes and 1082 co‐down‐regulated genes were identified. DEGs, differentially expressed genes; HBV‐ALF, hepatitis B virus‐related acute liver failure

To identify the differentially expressed genes between HBV‐ALF and normal liver tissues, we perform differentially expression analysis on three data sets. In total, 1608 up‐regulated and 1527 down‐regulated genes were figured out in GSE14668 (Figure 2A and Table S1); 1700 up‐regulated and 1314 down‐regulated genes were identified in GSE38941 (Figure 2B and Table S2), and for GSE96851, 1809 up‐regulated and 1264 down‐regulated genes were found (Figure 2C and Table S3). After deleting duplicated genes and those lacking expression values of specific gene symbols, the co‐up‐regulated and co‐down‐regulated DEGs from GSE14668, GSE38941 and GSE96851 were used to create a Venn diagram (Figure 2D,E). We ascertained 1277 co‐up‐regulated and 1082 co‐down‐regulated genes in three data sets (Tables S4 and S5), which were for further enrichment analysis.

### 3.4 Function and pathway enrichment analyses

**Figure 3:**
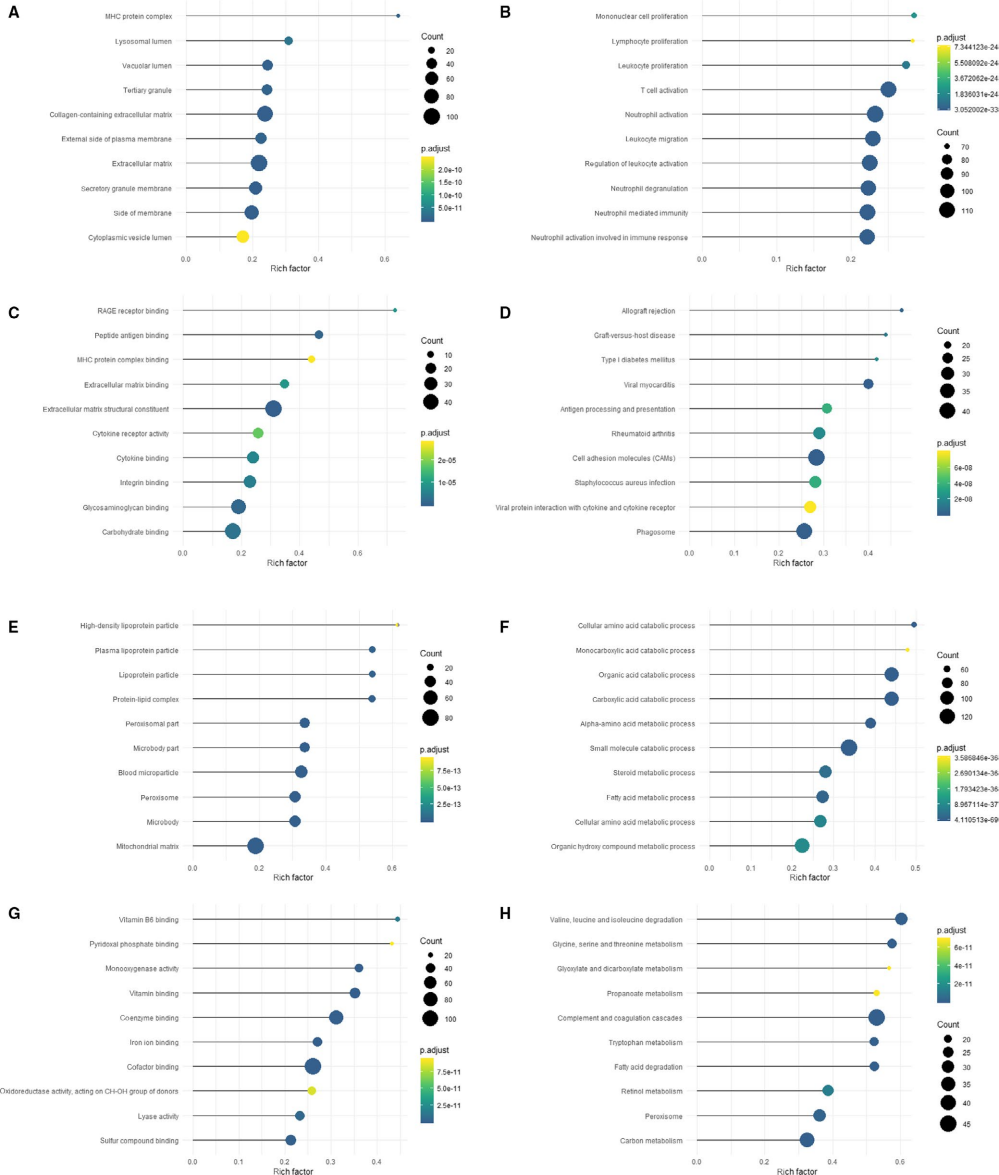
Functional enrichment analysis of co‐up‐regulated and co‐down‐regulated genes. A‐D, Top 10 CC terms (A), BP terms (B), MF terms (C) and KEGG pathways (D) of co‐up‐regulated genes. E‐H, Top 10 CC terms (E), BP terms (F), MF terms (G) and KEGG pathways (H) of co‐down‐regulated genes. CC, cellular components; BP, biological process; MF, molecular functions; KEGG, Kyoto Encyclopedia of Genes and Genomes

In order to explore the biological functions of the co‐expressed genes, we performed two kinds of enrichment analyses: (a) GO enrichment analyses and (b) KEGG enrichment analyses. Figure 3 showed the top 10 enrichment results of co‐up‐regulated and co‐down‐regulated genes. The co‐up‐regulated DEGs were enriched in the CC terms related to lysosomes and exosomes, extracellular matrix components (Figure 3A); those under BP terms were mostly associated with immune activation and inflammation response (Figure 3B), while those within MF terms were largely involved with intercellular communication and inflammatory protein binding (Figure 3C). The co‐down‐regulated DEGs enriched in the CC terms were basically concerned with lipid‐protein components (Figure 3E); those under BP terms were largely associated with amino metabolic and catabolic process (Figure 3F); simultaneously, those within MF terms were mostly in relation to vitamin function and binding (Figure 3G). As for KEGG pathway enrichment, the co‐up‐regulated genes were enriched in autoimmunity and infection immunity (Figure 3D), but the co‐down‐regulated genes were enriched in synthesis and metabolic function of various substances (Figure 3H).

### 3.5 Protein‐protein interaction (PPI) network and gene clusters analysis

**Figure 4:**
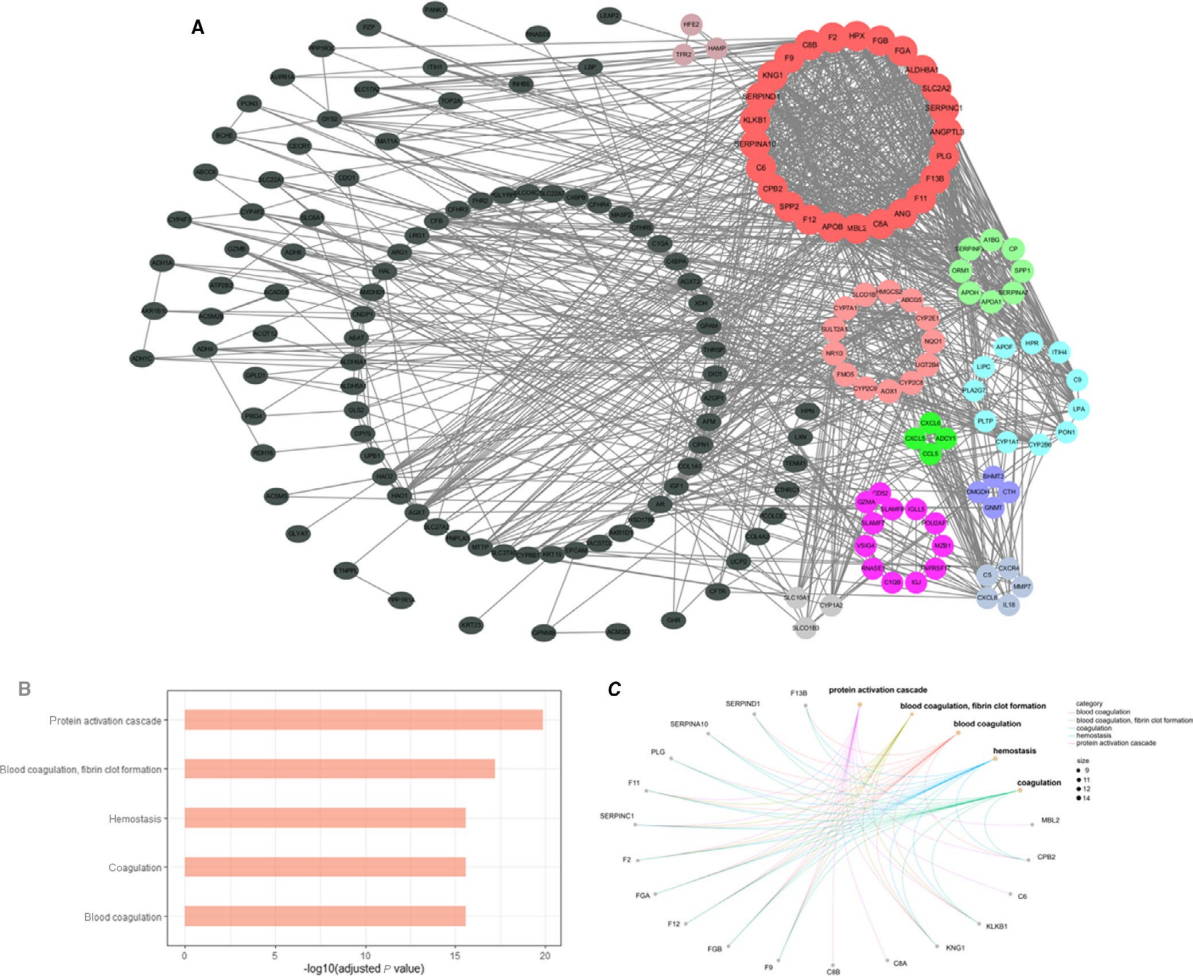
PPI network was processed by Cytoscape and MCODE plug‐in identified genes clusters. A, Genes cluster were noted with different colours except the black colour. The gene cluster with highest score was filled in red colour. B and C, The top 5 enrichment BP terms from the gene cluster with highest score. BP, biological process; PPI, protein‐protein interaction

To figure out the core genes from the DEGs in the HBV‐ALF samples, 217 genes with |log_2 _FC| > 3 and adjusted *P* value < .01 were uploaded to the STRING database for further analysis. A PPI network consisted of 208 nodes and 1141 edges was acquired (Figure 4A). Local clustering coefficient is 0.474 and PPI enrichment *P* value < 1 × 10^−16^. The result from STRING database was imported in Cytoscape for further analysis. The MCODE plug‐in was used to perform module analysis, and we obtained 10 clusters (Table S6). The genes from first cluster with highest score were all co‐down‐regulated genes and were selected for BP enrichment analysis from which we found that these genes were mostly enriched in coagulation (Figure 4B,C).

### 3.6 Immune infiltration analysis of HBV‐ALF

**Figure 5:**
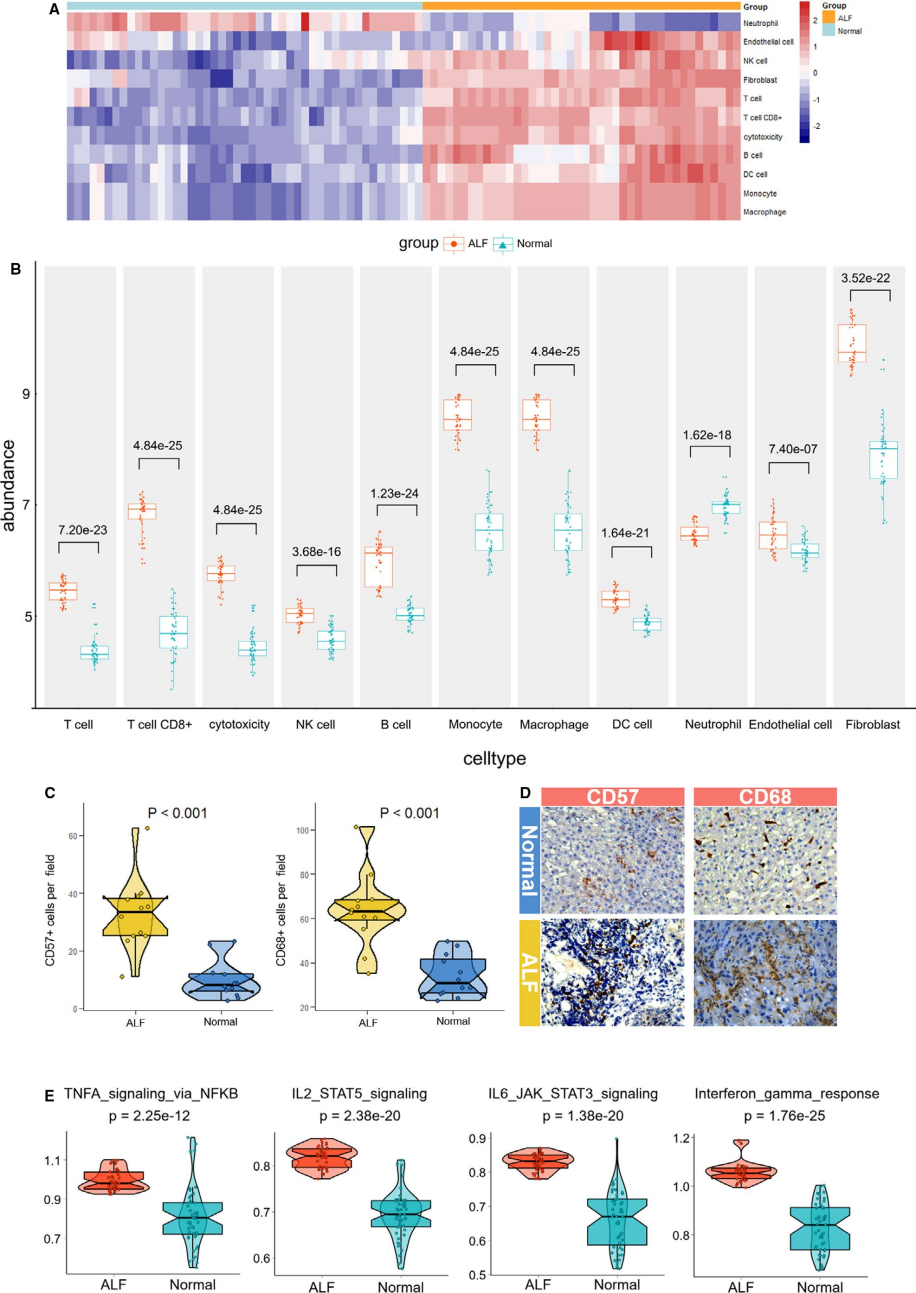
Immune microenvironment analysis of HBV‐ALF. A, Heatmap of 11 types of immune infiltration cells in HBV‐ALF and normal liver samples. B, The comparison of absolute abundance of 11 types of immune infiltration cells between HBV‐ALF and normal groups. C and D, The statistical analyses and micrographs showed the macrophages and NK cells infiltration in our HBV‐ALF and normal liver sample (n = 12 for each). E, Comparison of four important hallmark cytokine signal pathways between HBV‐ALF and normal groups. HBV‐ALF, hepatitis B virus‐related acute liver failure

To combine the three data sets (GSE14668, GSE38941 and GSE96851), we first used combat method to remove the batch effects. Principle component analysis of the gene expression profiles distinguished between HBV‐ALF and normal liver (Figure S1). Then MCP‐counter was employed to obtain the absolute abundance of 11 types of immune cell from each HBV‐ALF and normal sample. As shown in Figure 5A, HBV‐ALF presented strong immune cell infiltration. All immune and immune‐related cells except neutrophils showed a higher abundance in HBV‐ALF than normal liver (Figure 5B). Moreover, immunohistochemistry (IHC) detection of CD68 and CD57 further verified that HBV‐ALF patients had high infiltration levels of macrophages and NK cells (Figure 5C,D). In addition, ssGSEA showed four hallmark cytokine signal pathways were significantly activated in the HBV‐ALF than in normal liver (Figure 5E).

### 3.7 Identification of WGCNA modules and hub genes associated with HBV‐ALF

**Figure 6:**
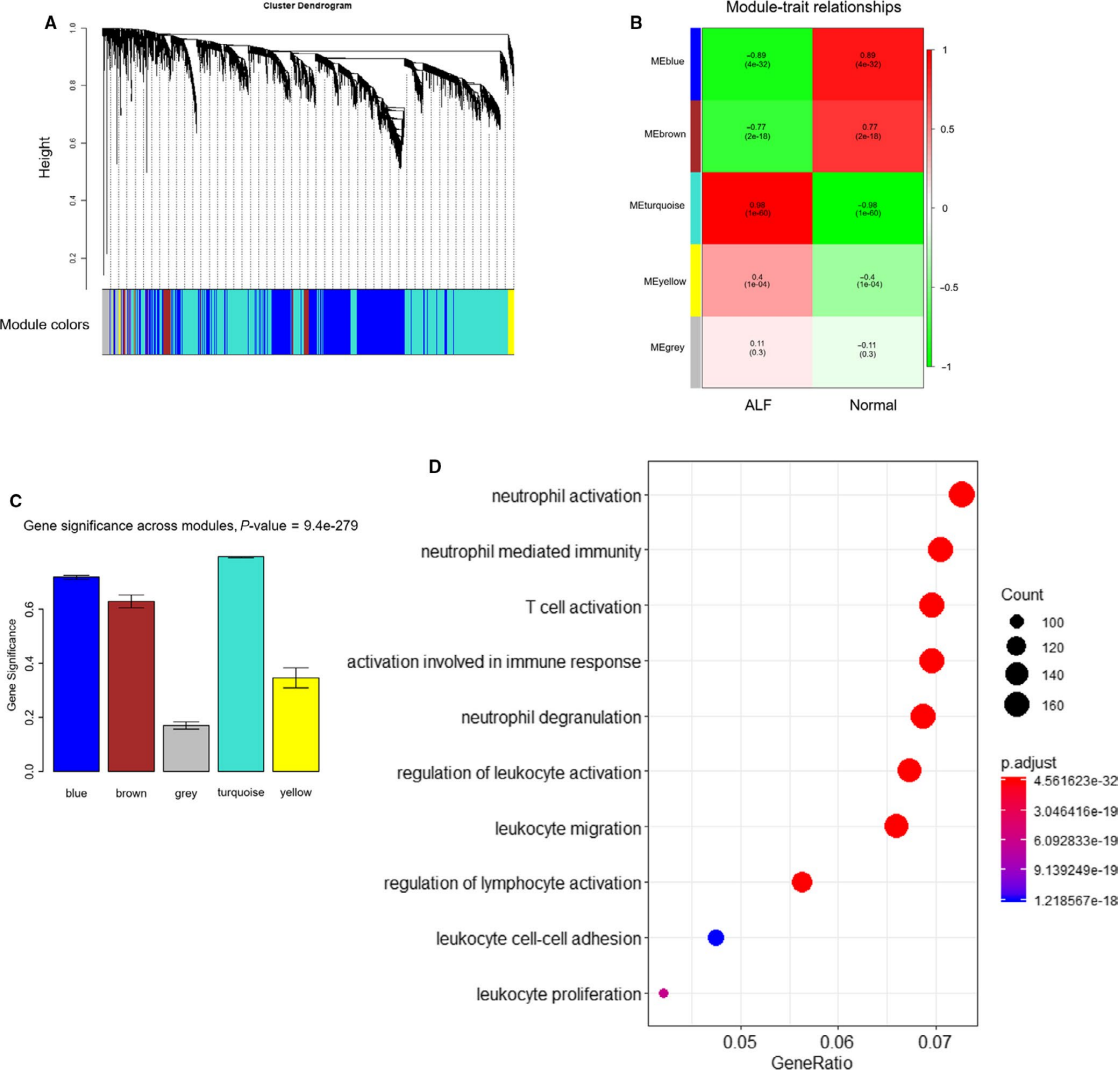
WGCNA of top 5000 median absolute deviation genes and identification of modules associated with HBV‐ALF. A, Hierarchical cluster tree showing four modules of co‐expressed genes. B, Heatmap of the correlation among module eigengenes, normal samples and HBV‐ALF samples. C, Distribution of average GS and errors in the modules associated with HBV‐ALF. D, The top 10 enrichment BP terms form turquoise module. BP, biological process; GS, gene significance; HBV‐ALF, hepatitis B virus‐related acute liver failure; WGCNA, weighted gene co‐expression network analysis

**Figure 7:**
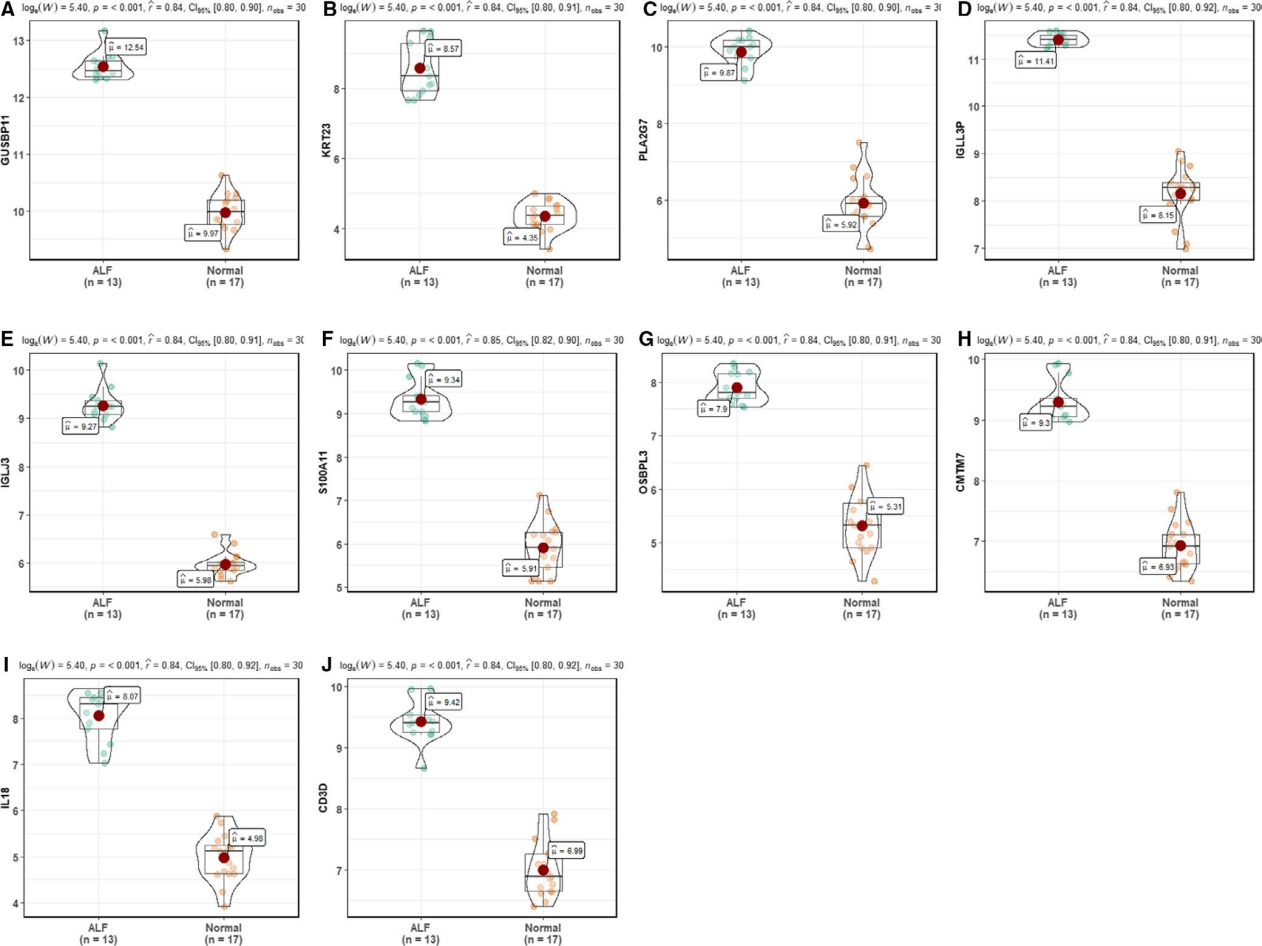
The comparison of hub genes expression based on an independent data set GSE62029. A, GUSBP11. B, KRT23. C, PLA2G7. D, IGLL3P. E, IGLJ3. F, S100A11. G, OSBPL3. H, CMTM7. I, IL18. J, CD3D

A weighted gene co‐expression network was conducted with the top 5000 median absolute deviation genes based on the combining expression matrix, resulting in identification of 5 WGCNA modules (Figure 6A,B). Module‐trait relationships analysis unravelled that ‘turquoise’ module of 2576 genes was highly related to HBV‐ALF (*r* = .98, *P* = 1×10^−60^) (Figure 6B). Besides, we found that the module significance of the ‘turquoise’ module was highest among the modules (Figure 6C). Therefore, the ‘turquoise’ module was considered as the key modules in our study. The genes from the key module were for BP enrichment and the top ten terms were shown in Figure 6D, which were also mainly associated with immune activation and inflammation response. The gene significance (GS) of genes form the key ‘turquoise’ module was calculated, and the top ten GS genes were regarded as key genes (Table S7). Furtherly, we compared the expression of these hub genes between the HBV‐ALF and normal samples from GSE62029. Expectedly, all these genes were up‐regulated in HBV‐ALF tissues (Figure 7A‐J), which suggested that these genes should play a significant role in the progression of HBV‐ALF.

## 4 DISCUSSION

In this study, the genetic profiles of HBV‐ALF and normal samples were compared to identify DEGs. Eventually, based on three data sets, 1082 co‐down‐regulated genes and 1277 co‐up‐regulated genes were selected. The enrichment analysis revealed that the co‐up‐regulated DEGs were mainly concentrated in the immune response. Several previous studies have evaluated the primary role of the immune response in the pathogenesis of ALF.^20^, ^21^, ^22^ Moreover, the significant role of inflammatory cytokines in liver injury has been identified in several studies,^23^, ^24^, ^25^ and anticipatedly many cytokine genes, such as GZMA, IL18 and IL16, were contained in the co‐up‐regulated genes. Besides, we calculated four important hallmark cytokine signal pathways by ssGSEA method, and expectedly, the TNF‐α, IL‐2, IL‐6 and IFN‐γ signal were activated in HBV‐ALF. Previous studies have demonstrated the significant influence of cytokines on hepatocyte regeneration, extrahepatic complications and hepatocellular death.^26^, ^27^ The enrichment analysis of the down‐regulated DEGs revealed that the down‐regulated genes were mainly associated with the substance metabolism and synthesis, which involved with amino, complement, coagulation protein and so on. PPI network and gene cluster analysis especially focused on the coagulation function. Therefore, the coagulation cascade pathways might be necessary in the pathogenesis of HBV‐ALF, which suggests that clinician should attach more importance to coagulation related issues while dealing with HBV‐ALF patients.

The focus of previous studies regarding HBV‐ALF is viral factors and virus‐host interactions,^28^, ^29^ in which immune cells, as the executors of the immune system, would undoubtedly play an important role. MCP‐counter found all the immune cells except neutrophils infiltrated significantly in HBV‐ALF liver compared with normal tissue. Neutrophils are short‐lived and will die quickly by apoptosis after responding to infection.^30^ We speculated that neutrophil abundance decreased because of mass death after fulfilling responsibility, which may cause more intensive inflammation response. Farci et al^9^ demonstrated that ALF resulting from HBV was mediated by an intrahepatic B cell response against the core antigen of HBV. In their study, massive IgG and IgM secreted by plasma cells accumulated in the liver show an unsubstituted role of humoural immunity in HBV‐ALF. As we all known, antibody‐dependent cellular cytotoxicity (ADCC) is a process of killing target cells depending on a variety of Fcγ receptors on the surface of cytotoxic cells and phagocytes, including the most important macrophages and NK cells.^31^ Our further IHC assay of CD68 and CD57 confirmed that macrophages and NK cells infiltrated more in HBV‐ALF clinical samples than normal liver. Mayumi Ishikawa et al confirmed that inherited immune attack of NK/NKT and macrophage cells lead to failure of liver regeneration.^32^ It also has been reported that macrophages and NK cells play a key role in the pathogenesis of ALF,^33^, ^34^, ^35^, ^36^ which is consistent with our findings. In addition to humoural immunity, inherited immune cells orchestrate the injury process in HBV‐ALF, which should be attached great importance.

WGCNA of the top 5000 median absolute deviation genes enabled us to identify a key ‘turquoise’ module of 2576 genes highly correlated with HBV‐ALF, which were largely enriched in immune‐related function. The top 10 GS genes in the key module, namely GUSBP11, KRT23, PLA2G7, IGLL3P, IGLJ3, S100A11, OSBPL3, CMTM7, IL18, CD3D, were selected as the hub genes, which were further confirmed significantly up‐regulation in an independent data set. It was previously reported that KRT23,^37^ S100A11^38^ and IL18^39^ contributed to liver injury. Besides, the serum levels of these three proteins could be detected easily. We have been suggested that they may represent specific markers to indicate and monitor the onset and progression of HBV‐ALF. As for the other hub genes, there was no previously relevant research in liver injury and ALF to the best of our knowledge; however, these genes may also contribute largely to HBV‐ALF.

In conclusion, for the first time, we systematically analysed the immune infiltration of HBV‐ALF based on multiple Gene Expression Omnibus data sets and we found fierce immune inflammatory response was the driving mechanism of HBV‐ALF. We then used IHC detection confirmed the importance of macrophages and NK cells infiltration in HBV‐ALF liver tissues. Besides, WGCNA revealed a key module that was also enriched in immune response. Ten hub genes were identified. The candidate genes and immune cells likely represent therapeutic targets for HBV‐ALF. However, bioinformatics only is not enough to explore the possible molecular mechanism of HBV‐ALF. Other functional experiments, including Western blot analysis, a luciferase reporter assay and gain‐ or loss‐of‐function studies, should be performed to verify our results. Thus, our follow‐up experiments will aim to perform additional experiments to unveil the mechanisms involved in HBV‐ALF.

## CONFLICT OF INTERESTS

The authors have no conflict of interests to declare.

## AUTHOR CONTRIBUTIONS

**Huadi Chen**, **Wenting Zhao** and **Yixi Zhang:** conceived and designed the study; **Weixin Luo**, **Xiaobo Wang** and **Changjun Huang:** searched databases and completed immunohistochemical experiment; **Huadi Chen**, **Wenting Zhao** and **Yixi Zhang:** analysed the data; **Yiwen Guo**, **Yu Nie** and **Maodong Ye:** prepared the tables and figures; **Huadi Chen**, **Dongping Wang**, **Maogen Chen**, **Qiang Zhao** and **Xiaoshun He:** wrote and revised the manuscript; all authors reviewed the manuscript.

## Supporting information

Fig S1

Table S1

Table S2

Table S3

Table S4

Table S5

Table S6

Table S7

## Data Availability

The data sets used and/or analysed in this study are available from the corresponding author on reasonable request.
