# Supplementary figures and images for: Systematic analysis on multiple Gene Expression Omnibus data sets reveals fierce immune response in hepatitis B virus‐related acute liver failure
Source: J Cell Mol Med. 2020 Jul 19;24(17):9798–809. doi: 10.1111/jcmm.15561 (PMC7520256; doi:10.1111/jcmm.15561)

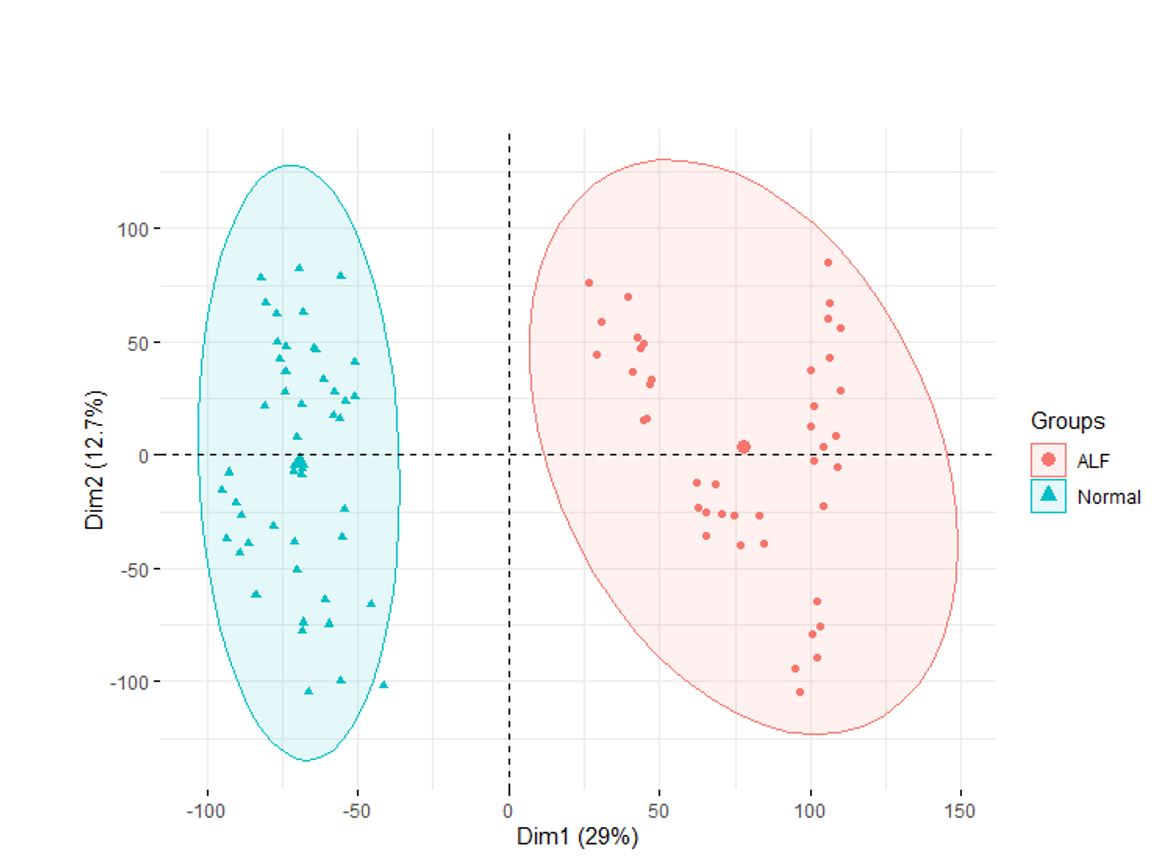

Supplement: Supplementary file 1 — Fig S1 [file JCMM-24-9798-s001.tif]
