# Supplementary material for: Systematic analysis on multiple Gene Expression Omnibus data sets reveals fierce immune response in hepatitis B virus‐related acute liver failure
Source: J Cell Mol Med. 2020 Jul 19;24(17):9798–809. doi: 10.1111/jcmm.15561 (PMC7520256; doi:10.1111/jcmm.15561)
Supplement: Supplementary file 7 — Table S6 [file JCMM-24-9798-s007.docx]

Table S6 Molecular Complex Detection(MCODE) plug-in was used to process the result downloaded from the STRING database to further identifying gene clusters. The clusters were presented as followed.

| Cluster | Score | Nodes | Edges | Node IDs |
| --- | --- | --- | --- | --- |
| 1 | 20.167 | 25 | 242 | MBL2, SPP2, CPB2, HPX, C6, PLG, ANG, SERPINA10, KLKB1, KNG1, C8A, C8B, SERPIND1, ANGPTL3, APOB, F9, FGB, F12, FGA, F2, SERPINC1, F11, ALDH8A1, F13B, SLC2A2 |
| 2 | 6.667 | 13 | 40 | FMO5, CYP2C8, SULT2A1, HMGCS2, CYP2E1, CYP7A1, NQO1, ABCG5, AOX1, NR1I3, SLCO1B1, UGT2B4, CYP2C9 |
| 3 | 6 | 8 | 21 | A1BG, CP, SERPINA7, ORM1, APOH, SERPINF2, APOA1, SPP1 |
| 4 | 5.4 | 11 | 27 | ITIH4, PLA2G7, LPA, CYP2B6, LIPC, APOF, PON1, C9, HPR, PLTP, CYP1A1 |
| 5 | 4 | 4 | 6 | CCL5, ADCY1, CXCL5, CXCL6 |
| 6 | 4 | 4 | 6 | BHMT2, DMGDH, GNMT, CTH |
| 7 | 3.636 | 12 | 20 | MZB1, GZMA, RNASE1, IGJ, IGLL5, POU2AF1, CD52, C1QB, SLAMF7, VSIG4, SLAMF8, TNFRSF17 |
| 8 | 3.5 | 5 | 7 | CXCL8, C5, CXCR4, MMP7, IL18 |
| 9 | 3 | 3 | 3 | SLCO1B3, SLC10A1, CYP1A2 |
| 10 | 3 | 3 | 3 | HFE2, TFR2, HAMP |
