# Supplementary material for: Systematic analysis on multiple Gene Expression Omnibus data sets reveals fierce immune response in hepatitis B virus‐related acute liver failure
Source: J Cell Mol Med. 2020 Jul 19;24(17):9798–809. doi: 10.1111/jcmm.15561 (PMC7520256; doi:10.1111/jcmm.15561)
Supplement: Supplementary file 8 — Table S7 [file JCMM-24-9798-s008.docx]

| symbol | GS | GSPvalue |
| --- | --- | --- |
| GUSBP11 | 0.9796489 | 1.84013E-62 |
| KRT23 | 0.9750971 | 1.08791E-58 |
| PLA2G7 | 0.9713627 | 4.38668E-56 |
| IGLL3P | 0.9700903 | 2.82997E-55 |
| IGLJ3 | 0.9680984 | 4.4831E-54 |
| S100A11 | 0.9675995 | 8.71308E-54 |
| OSBPL3 | 0.9642488 | 5.86879E-52 |
| CMTM7 | 0.9642468 | 5.88273E-52 |
| IL18 | 0.9632066 | 2.00388E-51 |
| CD3D | 0.9626029 | 4.0157E-51 |

Table S7 The top 10 GS hub genes

GS, gene significance; GSPvalue, gene significance P value.
